# Measuring quality of diabetes care by linking health care system administrative databases with laboratory data

**DOI:** 10.1186/1756-0500-3-233

**Published:** 2010-08-31

**Authors:** Helena Klomp, Roland F Dyck, Nirmal Sidhu, Paul J Cascagnette, Gary F Teare

**Affiliations:** 1 The Division of Quality Measurement and Analysis, Saskatchewan Health Quality Council, 241-111 Research Drive, Saskatoon, S7N 3R2, Canada; 2 Department of Medicine, Royal University Hospital, University of Saskatchewan, 103 Hospital Drive, Saskatoon, S7N 0W8, Canada; 3 Department of Community Health and Epidemiology, Royal University Hospital, University of Saskatchewan, 103 Hospital Drive, Saskatoon, S7N 0W8, Canada

## Abstract

**Background:**

Chronic complications of diabetes can be reduced through optimal glycemic and lipid control as evaluated through measurement of glycosylated hemoglobin (A1C) and low-density lipoprotein cholesterol (LDL-C). We aimed to produce measures of quality of diabetes care in Saskatchewan and to identify sub-groups at particular risk of developing complications.

**Findings:**

Prevalent adult cases of diabetes in 2005/06 were identified from administrative databases and linked with A1C and LDL-C tests measured in centralized laboratories. A1C results were performed in 33,927 of 50,713 (66.9%) diabetes cases identified in Saskatchewan, and LDL-C results were performed in 12,031 of 24,207 (49.7%) cases identified within the province's two largest health regions. The target A1C of <= 7.0% and the target LDL-C of <2.5 mmol/L were achieved in 48.3% and 45.1% of diabetes cases respectively. The proportions were lower among those who were female, First Nations, non-urban, younger and in lower income quintiles. The same groups experienced poorer glycemic control (exception females), and poorer lipid control (exception First Nations people). Among non-Aboriginal people, younger diabetic females were least likely to receive lipid lowering agents.

**Conclusions:**

Linkage of laboratory with administrative data is an effective method of assessing quality of diabetes care on a population basis and to identify sub-groups requiring particular attention. We found that less than 50% of Saskatchewan people with diabetes achieved optimal glycemic and lipid control. Disparities were most evident among First Nations people and young women. The indicators described can be used to provide standardized information that would support quality improvement initiatives.

## Background

Canadians are experiencing an epidemic of diabetes [1,2] that disproportionately affects First Nations people [3,4]. Although this has serious consequences for individuals, families and the health care system largely because of chronic complications, these can be lessened with improved glycemic and lipid control [5,6].

The Canadian Diabetes Association publishes clinical practice guidelines that include target values for glycosylated hemoglobin (A1C) and low-density lipoprotein cholesterol (LDL-C) [7]. We evaluated testing frequency and outcome measures of A1C and LDL-C indicators to produce population measures of quality of diabetes care and to identify sub-groups at particular risk for complications. This was achieved on a population basis through linkage of laboratory data with health care system administrative data.

## Methods

### Study Populations

This 2005/06 population based study of diabetes care in Saskatchewan was approved by the University of Saskatchewan, Saskatoon Health Region and Regina Qu'Appelle Health Region Ethics Review Boards. Study populations were identified from Ministry of Health databases used to administer health care to 99% of the provincial population [8]. Beneficiaries were sub-divided into First Nations people (FN) and other Saskatchewan residents (OSK) by age and sex. FN are indigenous to Canada and included those registered under Section 6 of the Indian Act [9]. Most OSK are of European origin but include non-registered FN (<0.5%) and Métis (about 5%) [10]. The provincial population was approximately one million people and >10% were FN [11].

Diabetic adults aged 20 years and older were identified using a validated algorithm [12] based on National Diabetes Surveillance System (NDSS) case definitions [13]. These required one hospitalization with a discharge diagnosis of diabetes (ICD-9 250.x or ICD-10-CA E10-E14.xxx), or two physician service claims for diabetes within any 730 day period. Gestational diabetes cases were excluded. We were not able to distinguish between types 1 and 2 diabetes but <2% of incident diabetes cases in Canada are <20 years [14], the group with the highest type 1 diabetes incidence [15].

We define the diabetes incident year as the first fiscal year (April1 to March 31) in which an individual meets the diabetes case definition when there has been no prior diabetes diagnosis for at least two years. Thereafter, for each year that an individual is covered by Saskatchewan Health, the individual is a prevalent case. Only prevalent cases of diabetes counted on the first day (April 1) of the 2005/06 fiscal year were included in this study; they comprised the core denominators for our analyses.

### Linkage of Diabetes Cases with Laboratory Data

Using encrypted unique identifiers, prevalent cases of diabetes were linked with laboratory data from centralized laboratories in the two largest Health Regions in Saskatchewan (Saskatoon and Regina/Qu'Appelle). This data accounted for nearly 100% of A1C tests and 50% of LDL-C tests performed in the province.

### Quality of Diabetes Care Indicators

Because this study predated the 2008 clinical practice guidelines [7], we used an A1C target of ≤7.0% for glycemic control and an LDL-C target of <2.5 mmol/L (now <2.0 mmol/L) for lipid control as published in 2003 [16]. A1C testing was recommended at diabetes diagnosis and then 3-4 times per year; LDL-C testing was recommended at diabetes diagnosis and then every 1-3 years as clinically indicated. Finally, we chose an A1C of >= 9.0% to indicate very poor glycemic control and a LDL-C of >= 2.5 mmol/L as a level when use of lipid lowering drugs should be considered.

Based on the above, the quality of care indicators were: proportion of diabetes cases tested for A1C in 2005/06; of those tested, the proportion with a most recent A1C <= 7.0%, a most recent A1C >= 9.0% and the mean of most recent A1C tests; proportion with >= 2 A1C tests/year; proportion with >= 3 A1C tests/year; proportion of diabetes cases with A1C <= 7.0% by frequency of testing (1, 2, 3, 4+ tests/year); proportion of diabetes cases ever tested for LDL-C in 2005/06; of those, the proportion with a latest LDL-C <2.5 mmol/L, and the mean LDL-C; and proportion of OSK diabetes cases with an LDL-C >= 2.5 mmol/L dispensed a lipid lowering drug (not available for FN). Overall results as well as by sex, ethnicity, location, age group and income quintile were calculated. Detailed descriptions of indicator derivations can be found in the Saskatchewan Health Quality Council 2008 *Quality Insight *report's Technical Appendix [17].

### Analyses

Most comparisons were made between groups within specific demographic sub-populations. Mean A1C and LDL-C values were compared using two tailed t-tests for two groups and one way Anova for multiple groups. For between group comparisons, we chose a reference group and calculated OR's with 95% confidence intervals for the remaining groups. Tests for linear trend (one sided and two sided) were used to compare within group proportions of diabetes cases with A1C <= 7.0% by frequency of testing. Data abstraction and analyses were carried out using SAS software version 9.1.3. A p value of <= 0.05 was considered significant.

## Results

Of the 50,713 prevalent diabetes cases in Saskatchewan during 2005/06 (Table 1), 24,188 were female (47.7%) and 6,184 were FN (12.2%). A majority (53.5%) were urban dwellers and 81% were >= 50 years. Almost half were in the lowest two income quintiles (48.3%).

**Table 1:** Outcomes of A1C testing among Saskatchewan adults with Diabetes by Demographic Characteristics

Characteristic	Number of persons with diabetes†	Tested for A1C		Number of persons tested	A1C <= 7.0%		A1C >= 9.0%		Mean A1C
					
		%	OR (95% CI)		%	OR (95% CI)	%	OR (95% CI)	
ALL SASK	50713	66.9		33920	48.3		12.6		7.4
female	24188	66.0	0.95(0.92-0.99)	15971	50.4	1.16(1.11-1.21)	12.3	0.95(0.89-1.01)	7.4^**1**^
male*	26525	67.7		17949	46.5		12.9		7.5
									
ETHNICITY									
FN	6184	59.0	0.72(0.68-0.76)	3646	40.6	0.80(0.74-0.86)	25.5	2.05(1.88-2.24)	8.0^**1**^
OSK*	44529	68.0		30274	49.3		11.1		7.4
									
LOCATION									
urban*	27147	68.4		18563	49.9		11.9		7.4
non-urban	23508	65.2	0.86(0.83-0.89)	15333	46.5	0.86(0.82-0.90)	13.6	1.23(1.15-1.31)	7.5^**1**^
									
AGE (years)									
20-29	686	51.2	0.55(0.47-0.64)	351	40.2	0.56(0.45-0.69)	24.5	4.26(3.31-5.49)	8.1^**1**^
30-39	2652	54.3	0.62(0.57-0.67)	1439	37.0	0.49(0.44-0.55)	27.4	4.96(4.33-5.67)	8.1^**1**^
40-49	6287	62.1	0.85(0.80-0.90)	3902	42.1	0.61(0.57-0.66)	20.6	3.39(3.06-3.76)	7.8^**1**^
50-59	10663	69.8	1.20 (1.4-1.26)	7443	44.4	0.68(0.64-0.72)	16.6	2.58(2.36-2.83)	7.6^**1**^
60-69	11596	72.5	1.37(1.30-1.44)	8408	48.1	0.79(0.74-0.83)	10.6	1.54(1.40-1.70)	7.4^**1**^
≥70*	18829	65.7		12377	54.3		7.1		7.2
									
INCOME QUINTILE									
lowest	12991	63.1	0.76(0.72-0.81)	8195	46.0	0.81(0.75-0.86)	15.7	1.53(1.38-1.69)	7.6^**1**^
second	11496	66.3	0.86(0.82-0.92)	7626	47.1	0.83(0.78-0.89)	13.4	1.31(1.18-1.45)	7.5^**1**^
third	8615	68.8	0.96(0.90-1.02)	5923	50.3	0.94(0.88-1.01)	10.9	1.06(0.94-1.19)	7.3^**1**^
fourth	7589	68.8	0.95(0.89-1.02)	5220	48.2	0.87(0.81-0.94)	11.3	1.08(0.96-1.22)	7.4^**1**^
highest*	9436	69.9		6593	51.2		10.7		7.3

All rates are crude rates

*Reference category

†Includes prevalent diabetes cases aged 20 years and older at beginning of fiscal year 2005/06

Values in this table are based on the last A1C result reported for year

^**1**^p < = 0.0001

Overall, 66.9% of people with diabetes had an A1C level done during 2005/06. Younger, female, non-urban people and those with lowest incomes were less likely to have an A1C test compared to their respective counterparts. However, the diabetic groups least likely to have an A1C test were those under age 40 (53.7%) and FN (59%). With the exception of females, the same demographic groups with fewest A1C tests also experienced the poorest A1C results. FN and those under age 40 exhibited the worst results with mean A1Cs of 8.0% and 8.1% respectively. The group with the best A1C profile were those aged 70+ (54.3% had A1Cs <= 7%, 7.1% had A1Cs >= 9% and the mean A1C was 7.2%).

Overall, 38.4% of adults with diabetes had >= 2 and 17.5% had >= 3 A1C tests during 2005/06 (Table 2). Results were similar between males and females. Non-urban people were less likely than urban dwellers to have >1 A1C tests during the year. However, those tested least frequently were FN.

**Table 2:** Frequency of A1C testing among Saskatchewan adults with Diabetes by Demographic Characteristics

	Number of persons with diabetes*	Persons with >= 2 A1C tests/year			Persons with >= 3 A1C tests/year		
		
		number	%	OR (95% CI)	number	%	OR (95% CI)
ALL SASK	48200	18256	38.4		8436	17.5	
female	23028	8767	38.1	0.99(0.95-1.03)	4000	17.4	1.00(0.95-1.04)
male†	25172	9759	38.8		4436	17.6	
							
ETHNICITY							
FN	5959	1747	29.3	0.69(0.65-0.74)	692	11.6	0.65(0.60-0.71)
OSK†	42241	16779	39.7		7744	18.3	
							
LOCATION							
urban†	25809	10351	40.1		4855	18.8	
non-urban	22338	8160	36.5	0.85(0.82-0.88)	3572	16.0	0.81(0.77-0.85)

All rates are crude rates

*Includes prevalent diabetes cases aged 20 years and older at beginning of fiscal year 2005/06 and who survived the year with SK Ministry of Health insurance coverage

†Reference category

Overall, the proportion of diabetic people with target A1Cs decreased from 49.1% of those tested once to 47.3% of those tested >= 4 times (Table 3). This downward trend was mirrored in males and OSK. Only FN experienced an improvement in target A1C by frequency of testing (38.9% of those tested once to 44.9% of those tested >= 4 times).

**Table 3:** Outcomes of A1C Testing by Frequency of Testing among Saskatchewan Adults with Diabetes

	Persons with only 1 A1C test/year		Persons with 2 A1C tests/year		Persons with 3 A1C tests/year		Persons with >= 4 A1C tests/year		Test for linear trend
		
	n*	% A1C <= 7.0	n†	% A1C <= 7.0	n†	% A1C <= 7.0	n†	% A1C <= 7.0	
ALL SASK	15034	49.1	10336	48.0	5371	47.5	3179	47.3	
female	7059	51.3	4864	49.8	2581	49.1	1467	49.7	p = 0.0259 (1 sided) = 0.0517 (2 sided)
Male	7975	47.1	5472	46.4	2790	46.0	1712	45.3	p = 0.0711 (1 sided) = 0.1422 (2 sided)
									
ETHNICITY									
FN	1866	38.9	1073	41.0	453	44.8	254	44.9	p = 0.0035 (1 sided) = 0.0070 (2 sided)
OSK	13168	50.5	9263	48.8	4918	47.7	2925	47.6	p = 0.0001 (1 sided) = 0.0001 (2 sided)

All rates are crude rates

* Denominator is all prevalent diabetes cases aged 20 years and older at the beginning of fiscal year 2005-06

† Includes prevalent diabetes cases defined at beginning of fiscal year 2005/06 and who survived the year with SK Ministry of Health insurance coverage

A1C values in this table are based on the last A1C result reported for year

Table 4 shows results of LDL-C testing among adults with diabetes in Saskatchewan's two most populous Health Regions. Of the 24,207 prevalent diabetes cases, 11,294 were female (46.7%) and 2,321 were FN (9.6%). Most were urban (77.3%) and the majority were aged 50+ (80.3%). Almost half (48.7%) were in the lowest two income quintiles.

**Table 4:** LDL Cholesterol Indicator outcomes among Diabetic adults in Two Saskatchewan Health regions by Demographic Characteristics

	Number of persons with diabetes†	Tested for LDL-C		Number of persons tested	LDL-C < 2.5 mmol/L		Mean LDL-C	
				
		%	OR (95% CI)		%	OR (95% CI)	Mmol/L	Significance
TOTAL	24207	49.7		12019	45.1		2.7	
female	11294	47.6	0.89(0.85-0.94)	5379	41.3	0.76(0.78-0.81)	2.8	p < 0.0001
male*	12913	51.4		6640	48.2		2.6	
								
ETHNICITY								
FN	2321	29.2	0.38(0.35-0.42)	677	47.9	1.26(1.07-1.47)	2.6	p = 0.19
OSK*	21886	51.8		11342	44.9		2.7	
								
LOCATION								
urban*	18702	57.1		10679	45.0		2.7	
non-urban	5476	24.1	0.23(0.22-0.25)	1322	46.2	1.03(0.92-1.15)	2.7	p = 0.18
								
AGE (years)								
20-29	358	29.1	0.49(0.39-0.62)	104	42.3	0.84(0.57-1.25)	2.8	
30-39	1379	35.1	0.64(0.57-0.72)	484	39.5	0.75(0.62-0.91)	2.8	
40-49	3040	46.8	1.04(0.96-1.13)	1423	42.1	0.82(0.73-0.93)	2.7	
50-59	5268	54.5	1.41(1.32-1.51)	2871	43.4	0.85(0.77-0.94)	2.7	p = 0.0002
60-69	5428	57.9	1.62(1.51-1.74)	3144	46.7	0.98(0.89-1.07)	2.7	
≥ 70*	8734	45.7		3993	46.9		2.7	
								
INCOME QUINTILE								
lowest	6170	45.8	0.74(0.69-0.81)	2826	44.1	0.91(0.81-1.02)	2.7	
second	5630	48.4	0.81(0.74-0.88)	2726	44.6	0.91(0.81-1.02)	2.7	
third	4499	49.9	0.85(0.78-0.92)	2247	45.1	0.93(0.82-1.04)	2.7	p < 0.02
fourth	4044	52.3	0.93(0.85-1.01)	2116	45.1	0.92(0.81-1.03)	2.7	
highest*	3770	54.8		2067	47.4		2.6	

All rates are crude rates

* Reference category

† Includes prevalent diabetes cases aged 20 years and older at beginning of fiscal year 2005/06 in Saskatchewan's two largest Health Regions

Overall, 49.7% of diabetic adults had at least one LDL-C test during 2005/06. Younger, female, non-urban people and those with lowest incomes were less likely to have an LDL-C test than reference groups. However, those least likely to have an LDL-C test were younger people and FN (29.2% versus 51.8% of OSK). Females displayed among the poorest LDL-C outcomes with only 41.3% achieving target LDL-C levels compared to 48.2% of males. Mean LDL-C was also poorer for females (2.8 mmol/L) compared to males (2.6 mmol/L). In contrast to A1C findings, FN and non-urban diabetic people were more likely to achieve target LDL-C levels than their OSK and urban counterparts.

During 2005/06, 41,934 diabetic OSK lived in Saskatchewan for the entire year. Of those, 36.9% were prescribed a lipid lowering agent compared to 38.6% of 20,714 diabetic OSK living in Saskatchewan's two most populous Health Regions. Table 5 shows that 6,914 diabetic OSK in the two Health Regions had a most recent LDL-C level >= 2.5 mmol/L, but only 39.2% of those received a lipid lowering drug. Although no significant differences were found by location and income quintile, there was a trend for better treatment rates with increasing income. The lowest treatment rates were observed in the oldest and youngest subjects, and among women. However, both males (27.0%) and females (18.8%) under age 40 were least likely to receive treatment.

**Table 5:** Use of Lipid Lowering Drugs among Diabetic OSK With LDL-C >= 2.5 MMOL/L by Demographic Characteristics

	Persons with LDL-C ≥ 2.5 mmol/L†	Persons dispensed a lipid lowering drug	Crude rate (%)	OR (95% CI)
TOTAL	6914	2711	39.2	
AGE (years) Female				
20-39	165	31	18.8	0.38 (0.26-0.58)
40-49	389	123	31.6	0.77 (0.60-0.98)
50-59	723	305	42.2	1.21 (1.00-1.46)
60-69	795	329	41.4	1.17 (0.97-1.41)
≥ 70*	1157	435	37.6	
				
AGE (years) Male				
20-39	152	41	27.0	0.72 (0.50-1.06)
40-49	428	174	40.7	1.34 (1.07-1.69)
50-59	978	442	45.2	1.61 (1.35-1.93)
60-69	1003	451	45.0	1.60 (1.34-1.91)
≥ 70*	1124	380	33.8	
				
LOCATION				
urban*	6196	2416	39.0	
non-urban	704	288	40.9	1.09 (0.9-1.3)
				
INCOME QUINTILE				
lowest	1544	569	36.9	0.92 (0.79-1.08)
second	1566	606	38.7	0.97 (0.83-1.13)
third	1320	515	39.0	0.96 (0.82-1.13)
fourth	1267	525	41.4	1.05 (0.90-1.24)
highest*	1191	482	40.5	

*Reference category

Drug prescribing data was not available for FN, hence, analysis for lipid lowering prescribing indicators was limited to OSK

Data are stratified by sex because age-sex interaction was present

† Sub-group of prevalent diabetes cases aged 20 years and older at beginning of fiscal year 2005/06 in Saskatchewan's two largest Health Regions

## Discussion

We measured A1C and LDL-C quality of care indicators in Saskatchewan's diabetic population by linking health care system administrative data with laboratory data. While over 2/3 of people with diabetes received A1C testing, less than 50% achieved target levels and 12.6% exhibited very poor glycemic control. Almost 50% of the study population received LDL-C testing but only 45.1% of those achieved target levels and only 39.2% of those with values above the target of 2.5 mmol/L were prescribed a lipid lowering drug.

Diabetic adults who were female, FN, non-urban, younger or in lower income quintiles were less likely to receive A1C and LDL-C testing. Apart from females, the same groups also experienced poorer glycemic control. While younger subjects and those with lower incomes also displayed poorer LDL-C results, the largest disparity was observed between sexes. Females had higher mean LDL-C concentrations and were less likely to achieve target LDL-C levels. Despite that, diabetic OSK females with sub-optimal LDL-C were the group least likely to receive a lipid lowering agent. This was particularly evident in younger women, so likely included individuals with type 1 diabetes. A somewhat unexpected finding was that FN exhibited better LDL-C profiles than OSK. In the context of overall evidence for poorer quality of diabetes care, this observation suggests that there may be underlying differences in lipid metabolism between FN and OSK [18].

The proportion of diabetic people achieving target A1C levels decreased with frequency of testing among OSK but increased among FN. The reasons behind the former finding are complex but possibly explained in part by more frequent follow-up care in those with more brittle or complicated diabetes histories. In contrast, the small numbers of FN with more frequent A1C testing likely represented a subgroup with better diabetes care. This suggests that the overall poorer glycemic control experienced by FN may be subject to improvement through closer monitoring.

Although some of the statistically significant differences found here (particularly for mean A1C and LDL-C) may not be clinically problematic, it is important to highlight that the poorest overall results with respect to frequency of testing and achievement of target values, occurred among diabetic FN people and were substantive. While not unexpected, these findings provide evidence for ethnic-based disparities in quality of diabetes care. This is reflected in higher rates of many diabetes complications including amputations, strokes and end stage renal disease, as well as in higher mortality rates among FN as we have recently shown using the same methodology [19]. Although the reasons behind these disparities are perplexing in the context of a universal health care system, a recent national survey of diabetes care in FN communities shows promise in elucidating at least some of the underlying problems by examining individual level factors [20]. Furthermore, these sobering findings and their causes are highly relevant to other indigenous and developing populations who are also experiencing disproportionate rates of diabetes and diabetes complications [21].

Strengths of this study include employment of a validated algorithm to identify diabetes cases [12], inclusion of total Saskatchewan populations, ability to carry out a linkage between laboratory and health care system administrative data, and the ability to sub-divide the population by ethnicity. A significant limitation in the use of secondary data to conduct diabetes research is the unavailability of important individual level health determinants such as smoking, hypertension, and obesity as well as the inability to determine the impact of acculturation on First Nations people. Other limitations included an inability to identify people of Aboriginal heritage other than FN, but this reduces the true differences between FN and OSK. Second, identifying cases using administrative data may underestimate the incidence and prevalence of diabetes [12]. Third, although we could not differentiate between types 1 and 2 diabetes, most Canadian adults with diabetes have T2DM [14]; the proportion is even higher for FN [15]. Nonetheless, it is likely that the A1C and LDL-C indicator results for younger people with diabetes are partly attributable to OSK with type 1 diabetes. Finally, there may be overlap between the demographic groups that we have described. In particular, poorer results for FN in A1C indicators may be partly related to younger age and lower incomes (or vice versa). However, that would not explain the poor results among young OSK (particularly females) regarding treatment with lipid lowering agents.

## Conclusions

Linkage of laboratory data with health care system administrative data can be used to provide standardized information for people with diabetes on a population basis that can be used to identify deficiencies in care and support quality improvement initiatives. The potential to do so exists throughout Canada and other jurisdictions with universal health care systems and/or electronic medical records serving large patient groups. For example, a recent study described the utilization and outcomes of A1C testing among people with diabetes in eastern Ontario [22]. Although their results were consistent with those reported here, they did not provide information for FN.

We have now shown that less than 50% of diabetes cases in Saskatchewan achieved optimal glycemic and lipid control and even smaller proportions were tested as frequently as recommended. Furthermore, disparities were most evident among FN who exhibited substantially lower testing rates and poorer glycemic control than others. The fact that significant gaps exist between evidence-based guidelines for diabetes care and their implementation is a cause for concern. These findings should provide an impetus for understanding those gaps, and for developing and monitoring new strategies designed to improve the quality of diabetes care.

## Competing interests

The authors declare that they have no competing interests.

## Authors' contributions

HK conceived the study, acquired the data, participated in its design and coordination, interpreted the data and helped to draft the manuscript. RD helped to conceive the study, participated in its design and coordination, interpreted the data and drafted the manuscript. NS and PC participated in the design of the study and performed the statistical analyses. GT participated in the design of the study, oversaw and coordinated the analyses, and interpreted the data. All authors read and approved the final manuscript.
